# Supplementary figures and images for: ColEval: Honeybee COLony Structure EVALuation for Field Surveys
Source: Insects. 2020 Jan 5;11(1):41. doi: 10.3390/insects11010041 (PMC7023294; doi:10.3390/insects11010041)

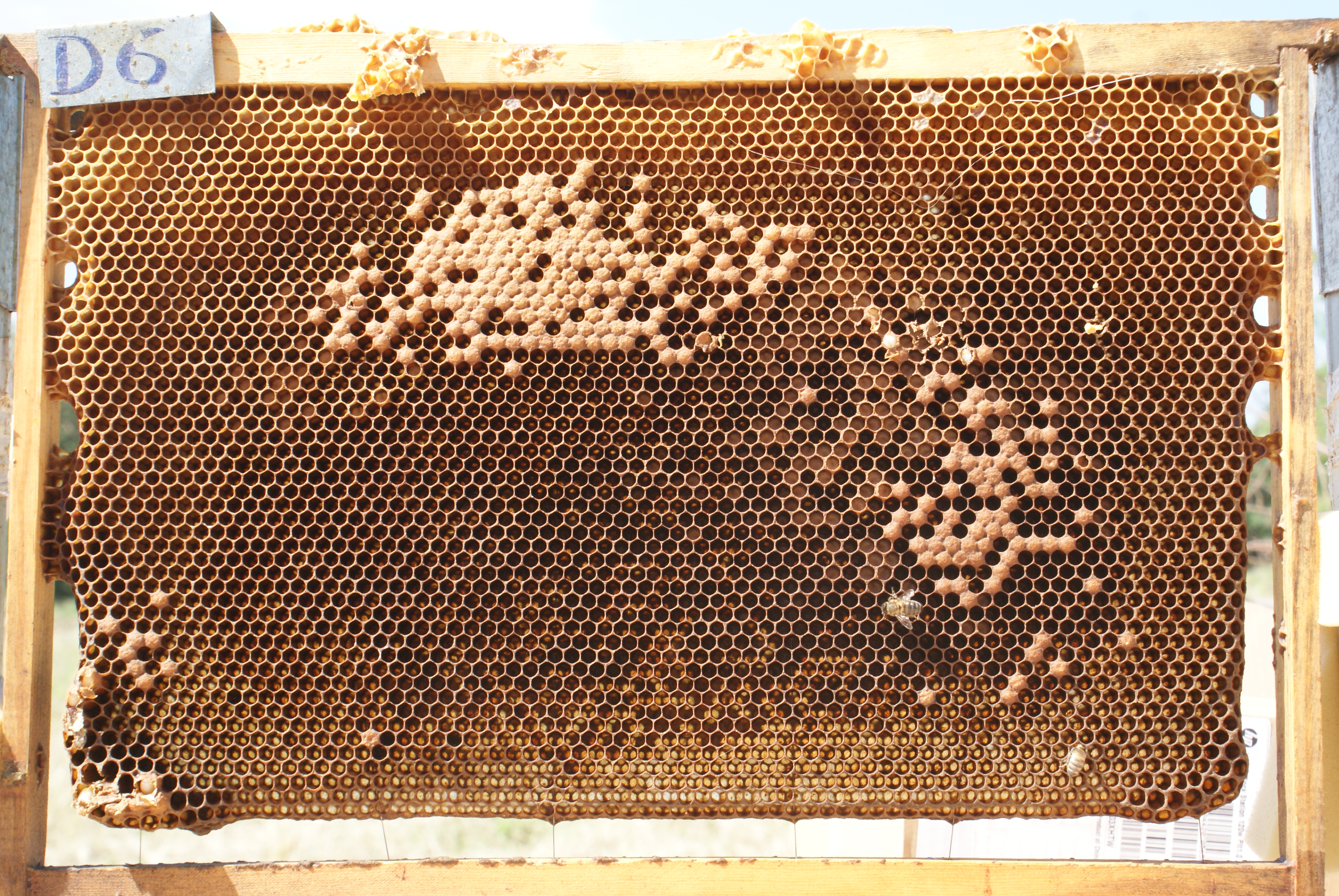

Supplement: Supplementary file 1 [file insects-11-00041-s001.zip › SM/SM3_BroodComb.JPG]
